# Quality of life of people with mental health problems: a synthesis of qualitative research

**DOI:** 10.1186/1477-7525-10-138

**Published:** 2012-11-22

**Authors:** Janice Connell, John Brazier, Alicia O’Cathain, Myfanwy Lloyd-Jones, Suzy Paisley

**Affiliations:** 1 Health Services Research, School of Health and Related Research, University of Sheffield, Sheffield, UK; 2 Health Economics, School of Health and Related Research, University of Sheffield, Sheffield, UK; 3 Information Resources, School of Health and Related Research, University of Sheffield, Sheffield, UK

**Keywords:** Quality of life, Mental health, Well-being, Ill-being, Functioning, Outcomes

## Abstract

**Purpose:**

To identify the domains of quality of life important to people with mental health problems.

**Method:**

A systematic review of qualitative research undertaken with people with mental health problems using a framework synthesis.

**Results:**

We identified six domains: well-being and ill-being; control, autonomy and choice; self-perception; belonging; activity; and hope and hopelessness. Firstly, symptoms or ‘ill-being’ were an intrinsic aspect of quality of life for people with severe mental health problems. Additionally, a good quality of life was characterised by the feeling of being in control (particularly of distressing symptoms), autonomy and choice; a positive self-image; a sense of belonging; engagement in meaningful and enjoyable activities; and feelings of hope and optimism. Conversely, a poor quality life, often experienced by those with severe mental health difficulties, was characterized by feelings of distress; lack of control, choice and autonomy; low self-esteem and confidence; a sense of not being part of society; diminished activity; and a sense of hopelessness and demoralization.

**Conclusions:**

Generic measures fail to address the complexity of quality of life measurement and the broad range of domains important to people with mental health problems.

## Introduction

There has been a shift in mental health services from an emphasis on treatment focused on reducing symptoms, based on a narrow notion of health and disease, to a more holistic approach which takes into consideration both well-being and functioning [1]. Mental health services in the United Kingdom, for example, are now being planned and commissioned based on psychological formulations addressing a person’s wider well-being, need, and functional outcome alongside, or sometimes in place of, diagnostic categories and clinical ideas of cure and outcome [2]. At the same time, there has been an increasing use of generic measures of health related quality of life like EQ-5D and SF-36 in assessing the benefits of health care interventions in order to inform decisions about provision and reimbursement (eg National Institute for Health and Clinical Excellence) [3] and for assessing patient reported outcomes [4]. It is claimed these generic measures are appropriate for both physical and mental health conditions; however some argue they are not suitable for people with severe mental health problems, particularly psychosis [5,6].

One of the challenges of using the concept ‘quality of life’ as a basis for outcome measurement is that it can be defined, and therefore measured, in innumerable ways. The assumptions underlying such measurement can be influenced by both academic discipline and ideological perspective [7]. As a result there are many different overlapping models of quality of life including objective and subjective indicators, needs satisfaction, psychological and subjective well-being models, health, functioning and social models [8]. One on-going tension is whether a measure should have a subjective or objective orientation. A subjective orientation may emphasise the importance of ‘being’, which in turn can be viewed either in hedonistic terms as the experience of current happiness or pleasure, or as a more eudemonic approach which considers the more pervading attributes of self-fulfilment, realisation or actualization [9,10]. A subjective evaluative approach may also be taken which asks people to rate how satisfied they are with their lives and aspects of it [11]. On the other hand, a more objective approach used in social policy places its emphasis on meeting needs, whether they are healthy, have sufficient income for food and satisfactory living conditions, are well educated and have access to resources [9,12]. A review of eleven instruments for measuring quality of life for people with severe mental illness identified that the most commonly assessed domains are employment or work, health, leisure, living situation, and relationships [13]. These measures combine an objective with a subjective approach that establishes levels of satisfaction with these different objective life domains. However, concerns have been raised regarding the limited coverage of domains assessed in such instruments [14,15]. Furthermore, it is criticised that measures have primarily been generated from the perspective of mental health professionals or other experts using a top-down approach rather than by an assessment of what individuals with mental health problems perceive to be important to their quality of life [15]. These are also important potential criticisms of the generic measures of health related quality of life like the EQ-5D and SF-36 [5].

The aim of this literature review was to examine the quality of life domains that are important from the perspective of an individual with mental health problems. This research was part of a larger project considering the applicability and suitability of generic health related quality of life measures for people with mental health problems (MRC project number G0801394).

## Methods

We sought to identify all primary qualitative research studies (involving methods such as interviews and focus groups) which explicitly asked adults with mental health problems what they considered to be important to their quality of life or how their quality of life had been affected by their mental health problems.

A range of approaches is available for synthesizing qualitative research [16]. Paterson et al. [17] recommend that the choice is made on the basis of the nature of the research question and design, the prevailing paradigm, and the researcher’s personal preference. In this review, framework synthesis was used. This is based on the ‘framework’ approach for the analysis of primary data [18] and is a highly structured approach to organizing and analyzing data which permits the expansion and refinement of an a priori framework to incorporate new themes emerging from the data [16]. It is appropriate here because the aim of our wider study was to identify whether existing outcomes measures are useful for measuring quality of life for people with mental health problems.

### Search methods

Systematic reviews of clinical effectiveness evidence require extensive searching based on a clearly focussed search question. Defining a focussed question was neither possible nor appropriate here because a pre-specified search question would have imposed on the search process an a priori conceptual understanding of the topic under review. Given the abstract nature of the relevant concepts and associated search vocabulary, and given the exploratory and inductive nature of the review process, we needed to use an iterative approach to searching. This incorporated a number of different search techniques including keyword searching, taking advice from experts, hand searching and citation searching of relevant references and world-wide-web searching. The iterative approach provided a means of accommodating within the search process new themes emerging from the review as the scope of our conceptual understanding developed. The identification of relevant search terms was an evolving process. Four search iterations were undertaken. The choice of search terms used in earlier iterations was based on our initial understanding of the review topic and on papers identified by experts at the outset of the review. The choice of search terms used in later iterations was informed by the review of evidence identified by earlier search iterations. Key terms included mental health; mental illness; mental disorder; quality of life; well-being; well being; life satisfaction; life functioning; life change; recovery; subjective experience; lived experience; lifestyle; coping; adaptation; qualitative; qualitative research. For a full list of search terms and details of the evolving search iterations see Additional file 1: Appendix 1 and Additional file 2: Appendix 2. Database searches were undertaken between October 2009 and April 2010 and included Medline, ASSIA, CINAHL, PsycINFO, and Web of Science. The searches were not restricted by date, language or country.

### Inclusion and exclusion criteria

#### Quality of life

The search started from a premise of not imposing a pre-conceived definition or model of ‘quality of life’. Whilst some studies retrieved had an explicit aim to explore quality of life we found other studies with very similar findings to those which explicitly examined the concept of quality of life even though quality of life was not the subject of investigation. These studies examined the concepts of: recovery, lived experience, subjective experience, psychosocial issues, health needs, and strategies for living. Complexities thus arose in deciding whether the studies were about the same substantive concept of quality of life or were tapping into a separate but overlapping concept. As Sandelowski [19] states ‘often research purposes and questions are so broadly stated it is only by looking at the kinds of findings produced that topical similarity can be determined’. We were aware of the danger that the inclusion of these studies could introduce themes that were not central to the concept of quality of life but were rather allied to a separate but related concept. A pragmatic decision was made to examine the research aims and interview questions of those studies which did not directly investigate the concept of quality of life and only include those which asked broad open-ended questions about how participants’ mental health affected their lives, what was important to or would improve their lives, or equated their findings with quality of life in some way. We excluded studies that deliberately started with a premise of the importance of any particular domain of quality of life or were structured solely around a pre-conceived list of domains.

#### Qualitative research

We included primary qualitative research studies that used qualitative interviews or focus groups data to identify the views of individuals with mental health problems. We excluded studies that used content analysis which presented results as a frequency list with no supporting participant quotes. Some studies sought the views of people with mental health problems and of carers or professionals; in such cases, we only included those studies in which the views of people with mental health problems could be separately identified.

#### Mental health

We included research on all mood disorders (eg depression, bi-polar, mania), neurosis and stress related disorders (eg anxiety, phobias, post traumatic stress disorder) personality disorders and schizophrenia, schizotypal and delusional disorders. Included studies had to state that participants had mental health problems as identified either through diagnosis, or through attendance at an establishment for people with mental health problems. Studies where mental health problems were secondary to a physical health problem were excluded.

#### Quality

The use of quality assessment in reviews of qualitative research is contested. Quality assessment is usually used in framework synthesis but this may be associated with its use alongside systematic reviews of effectiveness [16]. In this review, articles were not quality assessed and systematically excluded on the basis of quality. However, it was of paramount importance that any included study elicited the perspective of individuals with mental health problems and where this appeared not to be the case they were excluded. Consequently, studies were excluded when it was strongly suspected that the views of the researcher, or the method of analysis, had overly influenced the findings. These articles were examined and discussed at length by the research team before being excluded.

#### Language

**Figure 1:**
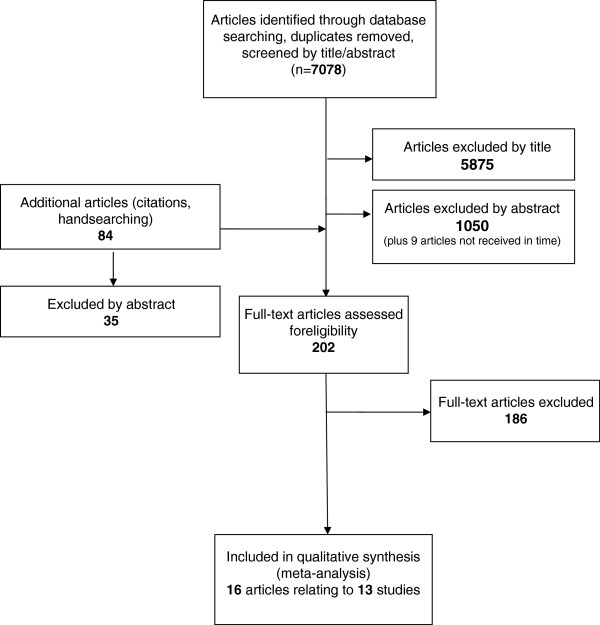
PRISMA flow diagram of searched articles.

Although the searches were not restricted to English language articles, non-English language articles were excluded because of the potential for mis-interpretation. Five potentially relevant articles were excluded on the grounds of language (Figure 1).

### Data extraction and analysis

The following details of the studies were extracted: mental health problem studied; author affiliation; time and location of study; number and demographic details of participants; research aims and questions; recruitment and sampling methods; and method of data collection and analysis. Themes within the findings and discussion sections were extracted for the thematic analysis.

Framework analysis [18] was used to allow the identification of common and variable patterns of themes within and across different studies. The first stage of framework analysis- familiarisation - was undertaken by reading all included papers. The second stage involved examining the findings from these papers to identify *initial* themes for a thematic framework. These ten initial descriptive themes were either identified as main themes from more than one study, or arose consistently across studies. These were: activity; relationships; the self; the future/aspirations; symptoms/well-being/emotions; spirituality; control/coping; insight/education; health care services/interventions; and resources/basic needs. The third stage, data organisation, involved charting data from the findings and discussion sections that corresponded to each theme. Text was transferred verbatim to ensure contextual accuracy. It was common for text to be identified as supporting more than one theme, for example a quote describing how work was good for their self-esteem would be placed in the thematic categories ‘activity’ and ‘self’. At the next stage each initial theme was examined and further sub-themes identified and documented within the framework chart. To assist with the final stage of framework - mapping - the sub-themes were listed and examined for their *conceptual* similarities and differences. To aid this process, we searched the wider literature to find papers which would help us to understand the data, to make connections between sub-themes, and to assist in the development of our final themes. For example, ‘belonging’ was an emerging theme, and we identified Hagerty et al’s [20] research which explored and defined this concept. We then returned to our framework chart to re-examine our data in light of the wider literature. Other influential literature was on the theory of ‘doing, being, becoming’ [21], ill-being vs well-being and intrinsic and extrinsic quality of life [22,23] and demoralization [24]. We have reported this literature when describing the theme in the findings because it was influential in shaping our understanding of the theme. The themes and domains from the included papers were presented and organised in contrasting styles by the authors of those papers. Depending upon the theoretical background of the researcher, and the method of analysis used, this resulted in themes which were either objective and descriptive (e.g. relationships, occupation) or abstract or metaphoric in their presentation (e.g. ‘Upset and calm changes patterns of being with and apart from others’). For the latter, whether a theme was major or minor was the subjective view of the authors. We have reported a theme as being a major theme within the studies if it was: a) a titled theme within the study findings b) was reported as being represented throughout the data or c) formed a substantive part of those studies that used abstract or metaphoric themes or of those that were not organised thematically. For transparency the original themes or section titles from the original papers have been presented after the quotes provided to illustrate our findings.

### Validation and trustworthiness

Validation procedures were incorporated into the review at all stages. Two researchers (JC and MLJ) independently identified articles from the first search iteration, and compared results to clarify the inclusion and exclusion criteria. Potential full articles were identified from further searches by the primary researcher and independently checked by the second researcher. The included articles were examined independently by both researchers to identify the main themes for the initial framework. Disagreements at all stages were resolved by discussion. Additionally, a multidisciplinary team of researchers met regularly in addition to meetings with clinicians and a user representative to discuss and challenge the inclusion and exclusion criteria, thematic framework, and conceptual interpretations and conclusions.

## Findings

### Description of included studies

Thirteen studies were identified from 16 articles [25-40]; two had fuller reports available, one an internal report [25,26] and the other a dissertation [27,28], the fuller reports [26,28] have been referenced in the findings. Further, one study indicated that not all emerging themes were presented in the paper and had a supplementary paper dedicated to the impact of bi-polar disorder on work functioning, which was included in our analysis [37,38]. The studies were published between 1994 and 2010 in a number of countries: Canada (5), UK (3), Sweden (2), USA (1), Australia (1) and New Zealand (1). The professional affiliations of the first author were occupational therapy (5), nursing (4), psychology (2), psychiatry (1) and social work (1). The mental health disorder most frequently represented was schizophrenia (or other psychotic disorder): this was the only population researched in three studies and the majority population in a further two. Three studies included individuals with bi-polar disorder only and one panic disorder only. Other studies had a mixed population including the above disorders plus persons with personality disorder, severe depression, and anxiety disorders. Two studies did not specify the disorder; they included persons described as having ‘enduring mental health problems’ and ‘psychiatric disability’.

Two studies had a primarily positive orientation in that they asked ‘what is required for a good quality of life’, and four studies a negative orientation through asking ‘how has your mental health affected your quality of life’. The remainder considered both ‘what had helped and hindered quality of life’. Most studies presented their findings descriptively, and four had a conceptual/abstract orientation. Further details of the studies [25-40] can be found in Table 1.

**Table 1:** Summary of included studies

**Reference number**	**First author**	**Year**	**Affiliation of first author**	**Country**	**Gender**	**Recruited from**	**Diagnosis**/**mental health problem**
[25,26]	Cook S	2009	Occupational Therapy	UK	M = 16	Community Mental Health Teams	17 x Schizophrenia; 4 x Bipolar; 4 x Other
					F = 8		
[27,28]	Corring DJ	2007	Occupational Therapy	Canada (Ontario)	M = 25	Mental Health Association Programs & Consumer Survivor agency	Schizophrenia n=27; Schizoaffective disorder n=7; Bipolar disorder n=4; Depression n=9; Posttraumatic stress disorder n=4; Anxiety disorder n=4; Borderline personality disorder n=4; Don’t know/no response; n=5
					F = 28		
[29]	Fisher MA	1998	Nursing	Canada	N = 24	Acute in-patient	Diagnoses included: Major depression; Bipolar; affective disorder; Depression/anxiety; Schizo-affective disorder; Psychosis; Sociopathic personality disorder
[30]	Gee L	2003	Psychology	UK	M = 3	Community Mental Health Teams/Acute inpatient	Schizophrenia
					F = 3		
[31]	Gould A	2005	Occupational Therapy	Canada	M= 4	via Poster	Schizophrenia or other psychotic disorder
[32]	Hamer HP	2009	Nursing - general and psychiatric	New Zealand	F= 10	Private psychology & psychotherapy clinics	Panic Disorder
[33]	Hedberg L	2009	Nursing/psychiatry	Sweden	M= 6	Out-patient	No diagnosis – ‘Psychiatric disability’
					F= 6		
[34]	Lalibertė-Rudman D	2000	Occupational Therapy	Canada	M= 25	Out-patient	Schizophrenia - DSM-IV diagnostic criteria
					F= 10		
[35]	Lim L	2004	Psychology	Australia	N= 18	Out-patient	Bipolar Disorder
[36]	Mayers CA	2000	Occupational Therapy	UK	M= 10	via O.T’s	No diagnosis - 'enduring mental health problems'
					F= 1		
[37,38]	Michalak E	2006	Psychiatry	Canada	M = 12	Outpatients and inpatient	Bipolar disorder
					F = 23		
[39]	Rusner M	2010	Social Work	Sweden	M= 6	Out-patient & advocacy group	Bipolar Disorder
					F= 4		
[40]	Vellenga BA	1994	Nursing	USA	M= 15	Out-patient	8 x Schizophrenia; 4 x Bi-polar; 3 x Schizoaffective

We identified six major themes: well-being and ill-being; control, autonomy and choice; self-perception; belonging; activity; and hope and hopelessness. The themes identified within each of the studies can be found in Table 2.

**Table 2:** Themes identified in research studies

	**25**-**6**	**27**-**8**	**29**	**30**	**31**	**32**	**33**	**34**	**35**	**36**	**37**-**8**	**39**	**40**
	**Mix**/**Sz**	**Mix**	**Mix**	**Sz**	**Sz**	**P**	**NS**	**Sz**	**BD**	**NS**	**BD**	**BD**	**Mix**/**Sz**
	**OT**	**OT**	**Nu**	**Pcl**	**OT**	**Nu**	**Nu**	**OT**	**Pcl**	**OT**	**Psy**	**SW**	**Nu**
				**Well-Being**/**Ill-Being**									
Distress/subjective experience of symptoms		**✓**		**✓**					**✓**				**✓**
Experience of psychosis/mania													
Fear/Anxiety/Worry													
Energy/motivation												**✓**	
Well-Being (positive concepts eg enjoyment/relaxation/stability)							**✓**						
Physical Well-Being													
				**Control**/**Autonomy**/**Choice**									
Control (general)								**✓**				**✓**					
Symptom control/management								**✓**	**✓**								
Information and understanding of illness								**✓**									
Choice (general)								**✓**									
Choice - related to limited finances								**✓**									
Choice -related to job opportunities								**✓**									
Independence/dependence																	
Personal strength, determination, self sufficiency																	
				**Self Perception**													
Self-identity/sense of self		**✓**							**✓**				**✓**				
Self-efficacy																	
Self-esteem																	
Self-acceptance/self-stigma																	
				**Belonging**													
Belonging/being part of the community							**✓**	**✓**									
Good relationships		**✓**	**✓**				**✓**					**✓**					
Support		**✓**					**✓**					**✓**					
Acceptance/ understanding		**✓**					**✓**										
Company/camaraderie/shared interests																	
Love, care and affection																	
Difficulties forming and maintaining relationships		**✓**	**✓**	**✓**													
Stigma		**✓**		**✓**							**✓**		**✓**				
Feeling normal		**✓**			**✓**			**✓**									
Loneliness/Isolation, alienation													**✓**				
				**Activity/Employment**													
Activity general		**✓**					**✓**	**✓**		**✓**							
Employment																	
Meaningful/enjoyable/suited to needs							**✓**										
Routine and structure											**✓**						
				**Hope and Hopelessness**													
Hope/hopelessness			**✓**						**✓**								
Goals/personal achievement																	
Loss and effect of past experiences			**✓**		**✓**				**✓**				**✓**				

**✓**Major theme Sub-theme.

*Mix*-mixed population; *Sz*-Schizophrenia only; *Mix*/*Sz*=Mixed with high proportion of schizophrenia; *P*-panic disorder; *BD*-Bi-polar Disorder; *NS*-not stated;

*OT*-Occupational Therapy; *Nu*-Nursing; *Pcl*-Psychology; *Psy*-Psychiatry.

### Well-being and Ill-being

Well-being has long been regarded as an important dimension of health related quality of life scales [14]. The emotional component of subjective well-being consists of high levels of positive affect (experiencing pleasant emotions and moods), and lack of low levels of negative affect (experiencing few unpleasant emotions and moods) [23]. Within our papers, symptoms of mental illness and aspects of emotional well-being were intertwined, with an emphasis on the negative rather than the positive. This suggested that ill-being, which is more akin to distress and the symptoms of mental illness, is an important aspect of quality of life for those with severe mental health problems.

The most evident ‘ill-being’ themes were general feelings of distress from symptoms; the experience of psychosis/mania; depressed mood; problems with energy and motivation and fear and anxiety.

#### Distress from symptoms

Distress, or the subjective experience of the symptoms of mental illness, was evident in the majority of studies [26,28,30-32,35,36,40] and a major theme in four [28,30,35,40]. The subjective experience of mental illness was described as wretched [36] a burden, debilitating, painful [40], tormenting [35], and as having a tyrannical power over life [28]. Pre-occupation with the symptoms of mental health problems interfered greatly with the most basic tasks of everyday living [26,28,31,40], making it difficult to deal with anything but the present moment [40]. Instead life was consumed with coping on a daily basis and living ‘one day at a time’ - sometimes on a moment to moment basis [28,31,34].

*Symptoms of mental illness were described primarily in negative and restrictive ways*. *Subjects reported continually trying to deal with the symptoms*, *describing symptoms as* “*a great burden*.” *The symptoms seemed to be so encompassing that these men had difficulty seeing beyond the pain of today*. “*This illness is a great burden*. *Day*-*to*-*day survival is a big question*, *and I just feel in a turmoil a lot of the time*”; “*I*’*ve had terrible suffering for over 20 years.”* [*40*-*A pervasive feeling of distress*]

#### Experience of psychosis/mania

Distressing symptoms reported included hallucinations and delusions (particularly hearing voices, thought disturbances and paranoia) [26,28,30], reality disorientation [28], mania, and hypomania [38], feelings of discomfort, weirdness or oddness [28], and irritability or agitation [30]. These symptoms could interfere directly with day to day living by having an effect on behaviour control [26,30,35,38], concentration, memory or decision making [26,30,31] and sense of self-identity [28,31,37].

“*When I hear voices erm*, *that stops me from doing a day to day existence*, *I*’*m preoccupied with the voices*”; “ … *the voices*, *how they*’*ve affected my life*, *erm*, *er just day to day living basically*… *Erm just er*, *getting out*, *getting out and doing things er*…*go to the shops*, *erm*, *erm*, *cooking*, *anything*, *anything like that*”; “*I daren*’*t go out now*, *thoughts in my head*, *make me think bad things*; *I get paranoid when there*’*s crowds of people.*” [*26*-*Fear of exacerbating mental health difficulties*].

#### Depressed mood

Depression was a diagnosis of a proportion of participants in two of the studies [28,29] and bi-polar disorder the primary diagnosis in three further studies [35,37,39]. Negative affect, in the more severe form of depression including feeling suicidal [26] (as opposed to simply being sad, unhappy), was also identified in studies where the primary diagnosis was psychosis related [26,30,34,36,40]. It was also the symptoms of depression in bi-polar patients that were reported as being particularly distressing [35], together with the unpredictability and instability of mood [35,38].

#### Energy and motivation

Depression was often expressed as associated with a lack of energy and/or motivation. Although energy and motivation might be regarded as two distinct concepts (physical and psychological), they were closely associated and for the most part reported together within the primary research. Energy, or lack of it, was a major theme in one study [39] and all but three of the primary research articles [32,33,35] described the debilitating effects of lack of energy. The three studies where energy and motivation were not evident focused on the nursing implications of panic disorder [32] the psycho-social issues related to bipolar disorder [35] and the positive determinants of health [33]. Participants reported feeling generally drained of energy [26,28-30,38,40] associated with a lack of motivation, enthusiasm, or interest in things [26,28-31,34,36,40]. The side effects of medication [26] or problems with sleep [26,30] were reported as having a causal effect.

“*The quality of my life in the last few years has been horrible*, *because it has taken so much energy and struggle to get through so many things*…. *I*’*ve got to get out*, *go out and do things or go to concerts or go to school things*, *or go to meetings or something*, *and doing some of those things is so tough*, *to make yourself*, *you know*, *get up and go*. *Just getting up to go out for a walk was really hard for me*, *whereas walking is one of my*, *you know*, *I love to go out and walk*.” [*29*-*Distant hopes fuel the relentless struggle to carry on*]

Because lack of energy was a problem, conserving energy for those activities that brought pleasure and joy was important [39]. Whilst lack of energy was the dominant theme, hypomanic states in bi-polar disorder were associated with increased energy and enthusiasm but were often short-lived with a return to a usual depressed state [38].

#### Fear and anxiety

Two studies reported that ‘fear’ was a theme that was represented throughout their interview data [28,30]. Fear, anxiety, or worry was present in some form in all of the studies. The subjective experiences of the symptoms were reported as being very frightening [26,28,31,32,35,40]. This tended to be identified in the studies on schizophrenia, bi-polar disorder and panic disorder. As a consequence, individuals lived in fear of relapse or a return to hospital [26,28,30]. There were associated financial worries which had implications for planning for the future and making commitments [30,34,37].

*Living day to day with a psychotic illness was described as a very frightening and isolating experience*. *The participants described their sense of fear while experiencing symptoms*, *watchfulness for reoccurrence of illness*, *concerns over safety*, *experiences of anxiety and rejection in interactions with others*, *avoidance of stressors*, *feelings that they were being treated as* “*fragile*” *by their families*, *and a sense of powerlessness in gaining control over symptoms* [*27*-*The experience of illness*]

Anxiety in social situations was especially evident and took various forms including anxiety about leaving the house, crowds and public places [26], concerns for their own safety [26,28,36], and that of others [26], worrying about what others thought of them and how they appeared [26,28,30], worries and concerns within relationships [29] and fears of rejection [40]. Worries concerning relapse or aggravation of symptoms and social anxiety often resulted in the avoidance of any activity or situation which might be perceived as stressful [26,28,30,33] thus limiting the possibilities of improving other aspects of quality of life.

*Avoiding situations they had previously enjoyed because of fear of how they would appear or that the stress associated with those situations would mean deterioration in mental health*: “*I*’*ve cut down on the sort of positions I get myself in*… *because of bad experiences in the past*…. *you just try less things with the fear that you*’*re going to get very ill again and go to hospital*” [*30*-*reduced control of behaviour and actions*]

#### Well-being

Within the studies reviewed there tended to be an emphasis on the absence of ill-being rather than the presence of well-being. However, the positive themes identified that were important to people included an overall sense of well-being [31,33,39], feeling healthy [31], peaceful, calm and relaxed [26,30,33,39], stable [30,35], safe [33,39] and free from worry and demands [33,39]. Enjoyment or happiness were not identifiable themes within the reviewed studies but were associated primarily with the need for activities to be enjoyable [31,36,39].

#### Physical well-being

Physical health was not a strong theme within the reviewed studies. The compounding effects of physical health problems were indicated in two studies [26,36] and physical health was listed as the second most important aspect of quality of life by participants in another [37]. A healthy lifestyle was considered beneficial which included exercise, avoiding drugs and generally taking care of oneself [26,28,33].

### Control, autonomy and choice

The importance of aspects of choice and control to quality of life was identified in eight studies [26,28,30,31,34,35,37,39] and was a main theme in three of these [30,34,35]. It was often discussed in the context of the availability of external resources which enabled choice and control, including medication and treatment, support, information and finances.

#### Symptom control

One of the most evident aspects of control was the management of the most distressing or pervading aspects of mental illness, particularly for those with psychosis related disorders [26,28,31-35,38,39]. Control was usually described as being achieved through medication [26,28,33-35,39]. Having control meant that individuals could move beyond ‘the all encompassing world of their illness’ [28] and instead attend to other important areas of their lives [28,31]. However, medication could also have a detrimental effect on quality of life through side effects, [26,28,30,34] feelings of dependency, [34,35] and fear of the consequences of not taking it [30]. It was therefore necessary to find the right medication to balance symptom management and side effects [26,28,32,33,35,39] as a means to a sense of well-being [28].

“*I*’*m on good medication*, *no symptoms*, *no side effects*. *I used to go through all the side*-*effects and symptoms and I don*’*t have anything now*. .*before that*, *I never really felt human*. .*I*’*m human*, *I*’*m flesh*, *you know like that in my mind and*, *it*’*s just a good feeling*. *I can*’*t explain how I was*, *used to be but since I*’*ve been on this medication I feel like a human*… *I don*’*t have any side*-*effects or anything or any problems*. .*I just take my pills and go*. *Like I feel like a human being*… *it*’*s just great*”; “*I think for me*, *apparently the most important one is just managing the illness*…*different medications*, *side*-*effects*, *knowing what they are*… *for me there*'*s been limited discomfort*” [*28*-*Experience of illness*-*gaining control*]

The concept of control was particularly important for those with bipolar disorder, and was related to an inability to control or pre-empt the onset of mood episodes or their behaviour [35,38] and to a need for stability [35].

Being informed and having an understanding and insight about the illness was considered to be important [26,28,32,34,39]. To achieve this it was important to have an accurate diagnosis [32,33]. This meant that people could receive effective medication [33], knew what to expect for the future [28,33,39] and could develop strategies to manage their illness and deal with it better [28,34]. This was regarded as a first step on the way to recovery [32] and improving quality of life [39].

#### Independence/dependence

There was a complex relationship between independence, dependence, and support. Both support [26,28,31,33,34,37,39,40] and independence [32,33,37], particularly financial independence [37] were regarded as being important for quality of life. Support helped people manage their illness, access resources, and increase their self-confidence [33]. However, it could also result in feelings of dependency [26,37] with a resulting loss of a sense of control and self-esteem [37]. Hence there could be a dilemma between wanting help and support and at the same time resenting it [29]. On the other hand, *choosing* to be dependent could enhance power and control [39]. Personal autonomy, finding the optimum balance between support and independence, was therefore important to quality of life [25,33].

“*I think that*’*s a big part of what I recognize now as quality of life is feeling I can take care of myself without being heavily dependent on a long*-*term basis on either the welfare system or my Dad*, *unless I*’*m choosing to do so for a specific reason*” [*33*-*Independence*: ‘*Or rather*, *not being independent*, *but not being dependent*]

Personal strength, determination, and self-sufficiency were also regarded as important [26,32,33,38]. It meant people were able to make use of available resources and develop self-help and personal coping strategies [26,28,32,37] which in turn promoted independence and a sense of control [28,32,37].

#### Choice

The concept of choice was most associated with the availability of financial resources [26,28,30,33,34,37] and with limited employment opportunities [26,28,30,34,38,40]. Having sufficient financial resources meant people could more readily have a healthy lifestyle [33], engage in activities that promoted well-being [26,28,30,33], facilitate the attainment of an optimum balance between dependency and independence [26,37], have a choice in their surroundings [26,28,34] and be able to plan for the future [30].

“*I*’*d have had more money if I*’*d stayed in the* [*job*] … *I*’*d have been able to board the animals and go on holiday*. *I would have been able to afford a bigger house maybe even have some help with some of my domestic tasks*. *yes*. *it*’*s limited my choices*”…“*Lack of control of your finances because what you get in benefits goes immediately what with all the things you have to pay out for*. *So you have to be very careful*… *That*’*s another sort of loss of control of part of your life which doesn*’*t make you feel very good about yourself*” [*30*-*Financial constraints on activities and plans*]

Also of value was being able to choose whether or not to take part in things (particularly social activities), [28,34] flexible work conditions, [38] when and with whom to disclose mental illness, [34] and choices associated with mental health services, workers and interventions [26].

### Self-perception

A number of aspects of self associated with quality of life were identified: self-efficacy - having a belief and confidence in your own abilities; self-identity - having a perception of self and knowing who you are; self-esteem - having a sense of self-worth and self-respect; and self-stigma - internalizing the negative views of others. These were linked to a further theme of self-acceptance. These self concepts were closely associated and used interchangeably within the studies reviewed making them difficult to differentiate. Aspects of the self and self-perception were a major theme in three studies [28,32,35] and were present in some form within all of the other studies except one [29] which had an abstract analytical style and only had undertones suggesting low self- esteem/image.

#### Self-identity

Problems related to self-identity, having a sense of self and ‘knowing who you are’ appeared particularly to be related to bi-polar disorder, schizophrenia, and panic disorder. The studies described difficulties with having a coherent sense of self, identity, and personality [31,32,35,37,39].

‘*when you end up in the hospital with a full*-*blown mania and you think that you*’*re a king and you*’*re screaming at the top of your lungs*… *trying to eat your hospital bed and*, *and*… *you don*’*t know how to deal with it or*, *or how to be*. *You don*’*t know how to become yourself again*. *You don*’*t know what happened to you*. *It*’*s like your identity has been changed*. *It*’*s like somebody hands you a different driver*’*s license and you*’*re like*, ‘*Well who is this person*?’ [*37*-*Identity*]

This loss of a sense of self necessitated a re-negotiation [31] or reclaiming [32] of self, based on self-acceptance, self-knowledge and understanding, [31,32,37,39] and relationships with reliable others [39]. Spirituality also had a role in achieving a sense of self [28].

#### Self-efficacy

This concept was expressed in the reviewed studies primarily as a lack of self-confidence, but also as feelings of inadequacy, uselessness, failure, an inability to cope, and helplessness [26,28,30-33,35,37]. Mental health problems were associated with a lack of confidence [26,31,32,35,38]. This lack of confidence limited day to day functioning and activities [26], and access to helpful resources [26] and affected choice and opportunities in employment [26,28,38] and relationships [26,28]. Bipolar disorder could be associated with an increase in self-confidence during manic episodes [38].

#### Self-esteem and self-acceptance

The theme of self- esteem includes the concepts of self-image, worth, value, and shame, and a view of the self as ‘defective’ [26,28,30,35-37,40]. It was primarily reported as a negative concept closely associated with loss of self-identity [37] and confidence [35]. Occupational activity was considered particularly important for self-esteem and status [28,36,38], as was the satisfaction gained from helping others [28]. However, the difficulties encountered in obtaining employment often resulted in a lowering of self-esteem [30,40]. A closely related concept to self-esteem was the positive concept of self-acceptance, [28,32,37,40] acceptance of the self as a person with an illness [32], or the belief that the illness did not represent everything that they were [28,37].

#### Self-stigma

The theme of ‘the self’ was closely related to the next theme of ‘belonging’, particularly through the concepts of ‘stigma’ and ‘feeling normal’ (see below). This inter-relationship is most evident in the concept of self-stigmatization, an internalisation of the negative views of others [28].

*Individuals living with severe and persistent mental illnesses suffer from a form of stigma* - *self*-*stigma* - *perhaps the most powerful of all stigmas as it affects the inner sense of self in very profound ways* . ‘*I stigmatize myself*. *I just have a very low self*-*image*. *I*'*m kind of hard on myself for not conducting myself the way I should be*…*not being as productive as I could be*. *It*'*s a reflection from general community*'*s perceptions of what this illness is all about*. […] [*27*-*Sense of Self*: *Self doubt*, *criticism*-*a barrier*]

### Belonging

The concept of belonging has been defined as the experience of integration and personal involvement in a system or environment at differing interpersonal levels. It can have two dimensions: ‘valued involvement’ - the experience of feeling valued, needed, accepted; and ‘fit’ - the person’s perception that his or her characteristics articulate with, or complement, the system or environment [20].

Of the primary research studies included in the review, one identified ‘connecting and belonging’ as being important to quality of life [34]. Others identified closely related main themes: being part of a social context [33], rejection and isolation from the community [35], a need for acceptance by others [40], social support [37], relationships [28], barriers placed on relationships [30], labeling and attitudes from others [30], stigma [28,30,37,40], alienation [40], detachment and isolation [30].

#### Relationships

Relationships were clearly central to the concept of ‘belonging’. These relationships included close connections with family and friends and also more casual relations with the local community, in the workplace, with service providers or with society at large. The complex nature of relationships and the positive and/or negative effects on quality of life were evident in all the primary studies.

The provision of support was a particularly strong theme, being a major theme in three studies [33,37,39]. Both practical [26,28,32,33,37,39] and emotional [26,28,32-34,37,39] care and support was identified as important to quality of life. This could be from family and friends [26,28,31,33,34,37,39,40] or peers and work colleagues [28,38,39]. Also important was the support received from professionals [26,28,32,33,35,39]. When families and professionals were unsupportive, quality of life declined [26,28,39].

“[.] *if you have schizophrenia or you have mental illnesses a lot of support helps*, *helps you get back on track*”; “*The support that they give me means a lot to me*. *I wouldn*'*t be where I am today without my family and my friends*. *They*'*ve supported me in every little way that they could*… *like my Mom will drive me to doctor*'*s appointments*… *just having my family in* [*name*] *living around me*… *I know that if*, *if I can*'*t get somewhere myself I can always rely on family members to take me*” [*28*-*Relationships with supportive family members*]

Within the reviewed studies the most predominant benefits of good and reliable relationships were to feel accepted and understood [26,28,33-35,37,40], and having company, camaraderie and shared interests [28-31,33,34,36]. Good relationships also satisfied the need for love, care, and affection [26,28,33,34,37], facilitated the experience of joy, fun, and happiness [29,33], someone to talk to/share problems with [26,28,29,33,39], to feel needed/helpful to others [28,30,33,39], to have people in whom one had trust and confidence [26,33,39] and who provided motivation and encouragement [33].

*Connecting with others and achieving a sense of belonging emerged as key to quality of life*: “*You need friends to be happy*…*you need affection*, *you need to be loved by people*, *or else you would never get ahead in life*. *You will always be miserable and unhappy*” [*34*-*Connecting and Belonging*]

Given the importance of others, their well-being was also important to the quality of life of the study participants [33].

Whilst relationships which satisfied the need to belong were important, difficulties forming and maintaining these relationships were evident [26,28-30,34-36,40]. These difficulties included problems and tensions within supportive long term relationships [26,28,29,35,37].

“*My Dad considers me a problem son*. *My mother thinking it*'*s going to be a bit of a problem*… *you*'*re not treated with the same kind of respect that you were before*… *you*'*re not given the same kind of credibility*…*it*'*s not*, *not quite the same*. *You don*'*t feel a part anymore*. *You*'*re separated*… *You*'*re not even part of your family*… *you don*'*t feel part of the community*; *I don*'*t feel part of anything*.” [*28*- *Negative reactions from family members*-*a barrier*]

Problems with relationships represented a complex multidirectional interaction between the person and society at varying interpersonal levels. This interaction involved the effect of the person’s illness when relating to others, other people’s subsequent reactions and attitudes to them, and the effect of those reactions and attitudes in further exacerbating symptoms of anxiety and depression and affecting the person’s perception of themself. Examples of the barriers experienced in connecting and relating to people included cognitive and thought disorders resulting in problems with concentration and attention [28,30,40] problems controlling behaviour [30,35,37] including acting out [30,37], irritability, volatile or inappropriate behaviour [37], grandiosity or self-inflation [37], and feelings of anxiety when talking to or being around people, including problems with trust and paranoia [26,28,38].

#### Stigma

Stigma can be defined as ‘any condition, attribute, trait or behaviour that symbolically identifies the bearer as culturally unacceptable or inferior’ [41]. Stigmatisation was a major theme in four of the studies [28,30,37,40] and evident in three others [26,34,35]. The experience and perception of negative reactions on the part of family, friends, service providers, employers, and society at large was shown to have a detrimental effect on quality of life. Individuals felt that they were perceived as lesser human beings who were discriminated against and treated accordingly [28] and that they were feared, avoided, or not accepted, which in turn led to feelings of rejection, marginalization, or being written off [28,30,35,37,40]. As a result, disclosure of mental illness was problematic and often avoided, and this had consequences for employment and close relationships [28,34,37]. Stigma had a detrimental effect on most aspects of life, including relationships [26,28,30,37], employment and career [26,28,30,37], going out and pursuing leisure activities [26,30], obtaining services [28], and planning for the future [28]. Stigma was considered to be more predominant in bipolar than unipolar depression [37].

#### Feeling normal

A major barrier to achieving a sense of belonging was that informants were not perceived by others – and often did not perceive themselves – as “normal” [34]. Whilst feeling normal was something they held in high regard, instead they were aware of being perceived differently and consequently treated differently [40]. Feelings that they were different, and attempts to appear normal, do normal things, or be accepted as normal, formed a theme that permeated many of the studies reviewed [28,30,31,34,35,40], being a major theme of three [28,31,34]. This is consistent with the dimension of ‘fit’ within the concept of ‘belonging’ - the person’s perception that his or her characteristics articulate with, or complement, the system or environment [20].

…*most informants expressed a need to both feel and be perceived as normal*. *For example*, *Informant 2 remarked*, “*The thing is that I want to be a normal person and achieve something in my life*,” *and Informant 25 stated*, “*I*’*d like to be treated as equal in society*.” *Informants spoke about not feeling like other persons and implied that this set them apart*. *As Informant 16 stated*, *I don*’*t want to be mentally ill*, *I wanna be normal so I can study normally*, *go to school normally*, *get married*, *this and that*. […]” [*34*-*Connecting and belonging*: *being normal*]

#### Loneliness/isolation/alienation

Feelings of isolation, loneliness, and particularly the concept of alienation can be regarded as the antithesis of a sense of belonging. Whilst highlighted as a main theme in one study only [40], these feelings were evident within the themes of relationships and stigmatization in all studies except one [31]. The symptoms of mental illness, the barriers these caused in the formation of relationships, the stigma and consequential effects on the self, together with feelings of being different and not accepted, resulted in a pervasive sense of loneliness and isolation. People chose isolation, or avoided relationships, as a way of protecting themselves against rejection and dealing with the fears of how they appeared and what others thought of them [28,30,40]. The effects of being consistently treated as undesirable or different became internalised and further influenced their sense of self [28]. Isolation was further compounded by the feelings that they were the only person suffering in this way [28]. Hence, isolation was not just feeling as though they did not have any friends but became a painful feeling of despair that affected all aspects of life.

“*I think one of the things about schizophrenia*, *I don*'*t know whether it*'*s schizophrenia or whether it*'*s*, *it happens in other mental illnesses too*, *is this terrible*, *terrible kind of inner isolation feeling*, *like you*'*re the only person*…*who is going through what you are going through and you*, *and you*'*re completely alone* […] *it*'*s just a terrible*, *painful sense of utter loneliness and isolation*.” [*28*-*The Tyranny of Psychosis*-*a barrier*]

For quality of life, people wanted a reciprocal relationship with others [33,37] which involved understanding and acceptance [26,28,33-35,37,40]. This could be achieved through ‘supportive own’, those who share their illness and experiences [28,33,34,37,39], or through belonging to a religious community [26]. However, it was also possible to have a sense of belonging to a social network that was ultimately not beneficial to quality of life [37], and difficulties disentangling ‘real’ spiritual experience from hyper-religiosity when hypo/manic could make belonging to a religious community problematic [37].

### Activity

By ‘doing’, a person achieves a sense of self, mastery, and successfully participates in the external world [21,42]. The importance of activity in some form to quality of life was expressed in all of the studies except one (which examined panic disorder from a nursing perspective) [32]. There was a difference in emphasis between studies: some focused specifically on the benefit of employment [30,35,38,40] and others on activity or occupation in its broader sense, including both employment and leisure activity [26,28,31,33,34,36,39]. Whatever the type of activity, it was stressed that it should be meaningful or fulfilling [26,28,31,33,34,36,39], enjoyable, [31,36,39] and suited to need and capabilities [26,31,33].

The benefit of activity is that it can provide the means for many of the factors important to quality of life discussed above. It is through activity that the opportunity arises to interact with others and hence develop a sense of belonging [34]. Activity can also improve mood [26,28,31,33,34], increase energy and/or motivation [28,34,39], relieve stress [26] and boredom [34,36] and provide a distraction from problems [26,33,34]. It also helps self-esteem and self-confidence, engenders a positive self-identity, [26,28,30,34,36,38,40] and enables people to take control of their lives [34].

One further factor is how activity provides order, routine, and structure [30,33,34,37,39]. Routine and structure can be achieved through employment [30,37], childcare [30,37] or activity in general, be it work or leisure [33,34,37,39]. However, one study highlighted how too much structure could be problematic and that what was important was flexibility and choice [37]. Having a physiological routine - particularly regular sleep, meals, and exercise - was considered important for general well-being [37,39].

*Positive outcomes that could be derived from the strategy of using activity to structure and fill time included increased motivation*, *diversion from present problems*, *and avoidance of negative moods*: [.] “*The actual work*, *whatever it is*, *is good for the mind and soul*… *you forget yourself*. *You forget your own problems when you are working*” [.] “*In the morning I have to do something*. *Some job or something I should do*. *Otherwise*, *I become bored and then become depressed because I don*’*t have anything to do*…. *when I have nothing to do I become sad and unhappy and become very depressed*, *and I don*’*t know what to do*. *It is very difficult*.” [*34*-*Managing time*].

Whilst activity was almost universally considered to be beneficial, taking part could be difficult if the activity was too demanding and not suited to needs [33,38,39]. The symptoms of mental illness could make difficult even the most rudimentary of activities, such as self-care, cooking and shopping, [26,36] and taking up employment was especially problematic [31]. Even potentially enjoyable leisure activities were avoided because of concern regarding other people’s reactions, [26,30,36] problems relating to people, [36] and the associated fear and stress resulting in a deterioration in health [26,30]. Lack of money also put a restriction on enjoyable pastimes [30].

For those who were employed, interpersonal relationships at work were particularly affected due to social withdrawal and irritability, or interfering, inappropriate, or volatile behaviour during hypomania, although work productivity could increase during hypomania [38].

### Hope and hopelessness

Integral to the concept of hope is having dreams, goals and a positive view of the future. The importance to quality of life of having dreams and goals or personal achievement was evident in six of the studies [28,29,31,33,34,37], the importance of activity and/or life in general being fulfilling and having some meaning and purpose was also evident [28,33,34,37,40]. Both having dreams and goals and having meaning and purpose in life were necessary to instigate change, make plans, and to move forward. Again, the difficulty of achieving this was stressed [28,31,34]. Losses experienced in the past affected the view of the future with a perception of reduced opportunities and choices [35] and diminished hopes and dreams, [29,31] particularly in the fields of employment [30,38,40] and relationships [29,40]. Loss and the effect of past experiences was a theme in seven of the studies,[26,28-31,35,38,40] and a major theme in three of these [29,31,35]. These losses included the loss of life roles generally, and more specifically the loss of work and career opportunities, relationship and the parental role, skills and ability, time, financial losses, and, ultimately, the loss of a sense of self and identity. Losses which had occurred in the past were perceived as a burden [28] with a pervasive sense of ‘something missing’ [40] which had long-lasting effects and made life a constant struggle [29,35,40]. Participants compared their own lives negatively with those of others [29,35,40], or with their own lives before illness struck [29,31], and all this brought about feelings of failure, of being cheated, and a sense of unfairness [35,38,40].

Past losses, including the loss of meaning and purpose in life, a sense of helplessness and inability to cope, all brought about a sense of hopelessness, necessitating a renegotiation and a lowering of aspirations and priorities [28,29,31].

The concepts of ‘hope’ and ‘hopelessness’ permeated the review studies [29,30,35,39,40] and formed a major theme for two [29,35]. Hopelessness was an expression of the view that life would never change for the better, and brought about a pervasive feeling of distress [40]. Conversely, hope provided a catalyst for change and a better life [39].

"*Well*, *my whole life feels problematic*, *I feel as if I*'*m not going anywhere*… *I know it sounds negative and I*'*m not really negative like this all the time*, *but you know*, *I find it hard*, *projecting myself into the future*, *and leading a happy life*. *I don*'*t think my life is very happy at the moment*, *it*'*s not very fulfilling*. *I haven*'*t got any real struggles at the moment*, *but it could be better*. *I don*'*t know if it*'*s because of the illness or the sort of person I am*…"; "*I don*'*t have hope that I*'*ll ever have a nice boyfriend*, *I don*'*t have any hope that I*'*ll get married*, *I don*'*t have any hope that I*'*ll work a full week*—*week after week after week*. *I don*'*t really have hope for stability*…" [*35*- *Bipolar Patients*' *View of Their Future*]

## Discussion

We identified six major themes associated with quality of life for those with mental health problems: well-being and ill-being; control, autonomy and choice; self-perception; belonging; activity; and hope and hopelessness.

Measuring quality of life for people with mental health problems is of interest currently because of concerns about the emphasis of mental health services on reducing symptoms. Yet our review identified the importance of distress and symptom control from the perspective of people with mental health problems. Amongst academic circles quality of life has confusingly come to be known as anything which is not clinical [14]. However, this review of the qualitative literature indicates that, when those with severe mental health problems are interviewed, the distress related to symptoms is integral to their quality of life, and in some instances seeing beyond this distress is difficult.

One of the strongest themes revealed by the review was a sense of belonging achieved principally by good quality relationships and lack of stigma. It has been stated that people are fundamentally motivated by a need to belong [43], and that belonging is the missing conceptual link in understanding mental health and mental illness [44]. Our review also indicates that negative social relationships are detrimental to quality of life. This is supported by research that shows that, whilst a large social network and satisfaction with social relations are associated with a better quality of life [45], negative social interactions and stigma are related to a worse quality of life [46]. Social exchange theory emphasizes that social interaction entails both rewards and costs, and that negative social outcomes can have a greater impact on well-being than positive outcomes [47]. There is also evidence that loneliness is caused more by a lack of intimate connections than by a lack of social contact [48]. Hence, the important factor is the sense of belonging, rather than social contact. So, whilst there is a strong argument that those people who experience supportive, caring, loving relationships and have a sense of belonging have a better quality of life, it is less clear which is the more detrimental - to experience and risk the negative impact of uncaring and disrespectful relationships, rejection and stigma, or to protect oneself through self-isolation.

As good and poor relationships can have a positive or negative impact, so activity can both help and hinder quality of life. For some, the severity of symptoms can mean that basic self-care and day to day functioning are difficult. Activity beyond perceived capabilities can also result in feelings of anxiety, which in turn can lead to deterioration in other mental health symptoms such as hearing voices and paranoia. This results in avoidance of any potentially stressful situations. This finding is supported by the findings of research into the occupational activity of those with severe mental illness which indicated that, though employment was valued, people made choices constrained by fear of relapse, and entered, avoided, and shaped their social and occupational activity to remain well [49] It was found that doing too much could exacerbate symptoms, yet doing too little could also cause illness, and therefore people with severe mental illness sought out daily occupations with structure, flexibility, and easily met demands over which they had control [49]. Therefore, to achieve well-being and quality of life, people need to find a balance and be enabled towards what they are best fitted [50].

Although avoidance of social and occupational activity may reduce anxiety and the occurrence of other related symptoms, at the same time it can compromise other aspects of quality of life. The consequent reduction in choice and opportunity has a detrimental effect on self-esteem and confidence. However, self-worth is gained through positive social feedback and successfully engaging in activity. Lack of self-esteem has also been shown to increase the risk of psychiatric disorders, the development of delusions, and the maintenance of psychotic symptoms [51]. The perception of self is therefore both a cause and a consequence of mental health, and can therefore be regarded as being pivotal to quality of life.

In relation to the finding of the importance of hope and hopelessness to quality of life, parallels can be seen between the results of this review and the concept of demoralization [24,52] whereby a persistent inability to cope with internally or externally induced stresses result in feelings of helplessness, incompetence, and loss of mastery and control leading to diminished self-esteem, hopelessness and demoralization which in turn adds to the distress of symptoms and further reduces a person’s capacity to cope. The demoralized person clings to a small number of habitual activities, avoids novelty and challenge, and fears making long term plans [24,52]. This feeling of demoralization further impacts upon ill-being and, if untreated, leads to chronic distress and possible suicide [24,53].

### Strengths and limitations of review

The primary studies included those with severe mental health problems only, with a majority having schizophrenia or psychotic disorders. Where there was a mixed population, studies rarely indicated any differences between people with different diagnoses. The findings may therefore have biases towards those with psychotic rather than affective disorders. The evidence base could therefore be improved by undertaking research with a wider range of mental health conditions.

Findings from the primary studies could be negatively or positively oriented depending upon the approach: research that asked how the illness had affected quality of life led to negative concepts (e.g. fear/stigma/isolation) whereas research that asked what would improve participants’ lives resulted in positive concepts (e.g. love, support, understanding). Some research papers addressed both, and identified factors that both helped and hindered quality of life. There was a greater emphasis on negative than positive concepts in the primary studies, and this has influenced the analysis and subsequent findings.

The range of themes included in the reviewed articles was extensive, in this review we have focused on those that are most closely associated with ‘health related’ quality of life.

#### Setting boundaries

There were difficulties setting boundaries around themes because of the strong inter-relationship of the different domains which make up quality of life. To avoid repetition, sub-themes have been placed in the main theme with which they were considered to be most strongly associated, but aspects of these themes could be placed in other themes. For example, ‘feeling normal’ has been included under the main theme of ‘belonging’ but could also be regarded as an element of ‘ill-being/ well-being’ and ‘the self’. Likewise, symptom management through medication is also an aspect of ‘well-being’ but probably due to the emphasis on psychosis related disorders in the reviewed studies it was the control aspect of medication use that predominated.

Complexities also arose when setting boundaries around the concept of quality of life. It was evident that there was a considerable overlap in findings with studies examining ‘recovery’, ‘lived/subjective experience’, ‘psychosocial issues’, ‘health needs’, and ‘strategies for living’. After much discussion and deliberation within the team, these studies were excluded from the review. Since completing our analysis a systematic review of the concept of ‘personal recovery’ has been undertaken [54] a concept previously defined as ‘a way of living a satisfying, hopeful, and contributing life even with limitations caused by illness’ [55]. Interestingly, they identified five recovery processes comprising ‘connectedness’, ‘hope and optimism about the future’, ‘identity’, ‘meaning in life’ and ‘empowerment’ which are very similar to our own final themes. They do not include ‘well-being’ and this may be due to the rejection of an emphasis on symptoms within the recovery movement. This suggests that the concepts of ‘recovery’ and ‘quality of life’ are very closely related. This is important to understand as the concept of ‘recovery’ is gaining prominence as a guiding principle for mental health services [56].

### Implications for measuring quality of life

The findings of this review indicate six major themes associated with quality of life for those with mental health problems: well-being and ill-being; control, autonomy and choice; self-perception; belonging; activity; and hope and hopelessness. This provides important evidence for critically examining the content of measures currently being used in mental health and particularly the generic measures of health related quality of life like EQ-5D that are being used to inform resource allocation decisions and the monitoring of outcomes. Concerns with the generic measures have been that they are designed by experts with little or no input from people with mental health problems and their coverage is too limited. The EQ-5D, for example, has the following five dimensions of health: mobility, self-care, usual activities, pain and discomfort, and depression and anxiety. Respondents are asked to report their level of problems (no problems, some/moderate problems or severe/extreme problem) on each dimension to provide a position on the EQ-5D health state classification. A key concern raised about this measure is the focus on physical health rather than mental health problems [5,6]. These can be seen as a combination of physical functioning (mobility, self-care), well-being (depression and anxiety), social functioning (that may be included in usual activities) and physical symptoms (pain and discomfort). There is only a modest degree of fit between these EQ-5D dimensions and the six themes within our review. Anxiety and depression may reflect, however crudely, ill-being (though not well-being). Usual activity is again rather crude, but arguably covers aspects of activity. However it makes no allowance for the finding that some activity can have a negative as well as a positive impact. This leaves the themes of control, autonomy and choice; self-perception; belonging; and hope/hopelessness which are not addressed within the EQ-5D.

The findings of this review can help to provide useful evidence for examining the content validity of different measures. This evidence can be used alongside quantitative psychometric evidence on the performance of measures in different groups. In the case of EQ-5D, for example, recent reviews have found supporting evidence for construct validity and responsiveness in people with depression and personality disorder, but reflected the concerns about their appropriateness for those with anxiety, bipolar disorder and schizophrenia [57-59].

## Conclusions

A good quality of life is characterized by feelings of well-being, control and autonomy, a positive self-perception, a sense of belonging, participation in enjoyable and meaningful activity, and a positive view of the future. In contrast, a poor quality of life is associated with feelings of distress, lack of control over symptoms and life in general, a negative perception of self, stigmatization and rejection, diminished activity and difficulties with day to day functioning, and a negative outlook. These life domains interact in a complex and reciprocal way. Generic measures of quality of life may fail to address this complexity and the rich and broad range of domains important to people with mental health problems.

## Competing interests

The authors declare that they have no competing interests.

## Authors’ contributions

JC, primary researcher, screened titles and abstracts, reviewed included papers, conducted additional searches, developed the conceptual framework, analysed the data, and drafted the manuscript. JB, principal investigator, reviewed included papers, reviewed and advised on the conceptual framework. AOC, co-investigator, reviewed included papers, reviewed and advised on the conceptual framework. MLJ, co-investigator, screened titles and abstracts, reviewed included papers, and developed the conceptual framework. SP developed the search strategy, conducted the electronic database searches and drafted the related section of the manuscript. All authors reviewed and revised drafts and approved the final manuscript.

## Supplementary Material

Additional file 1 **Appendix I.** Summary of search iterations. Click here for file

Additional file 2 **Appendix II.** Keyword search strategies. Click here for file
