# Supplementary material for: Quality of life of people with mental health problems: a synthesis of qualitative research
Source: Health Qual Life Outcomes. 2012 Nov 22;10:138. doi: 10.1186/1477-7525-10-138 (PMC3563466; doi:10.1186/1477-7525-10-138)
Supplement: Additional file 1 — Appendix I. Summary of search iterations. [file 1477-7525-10-138-S1.doc]

**Appendix I : Summary of search iterations**

Medline using QoL terms

Medline using possibly related QoL terms

ASSIA, CINAHL, PsycINFO, WOS using QoL terms

Hand and citation searching

All databases using quasi QoL terms

Hand and citation searching

Studies identified through experts and web searching

No relevant

studies identified

**1^st^ search iteration**

**2nd search iteration**

**3rd search iteration**

**4th search iteration**

= search activity

= end of search activity

= link indicating results of one search activity informing scope of another search activity in next search iteration
