# Supplementary material for: Quality of life of people with mental health problems: a synthesis of qualitative research
Source: Health Qual Life Outcomes. 2012 Nov 22;10:138. doi: 10.1186/1477-7525-10-138 (PMC3563466; doi:10.1186/1477-7525-10-138)
Supplement: Additional file 2 — Appendix II. Keyword search strategies. [file 1477-7525-10-138-S2.doc]

**Appendix II : Keyword search strategies**

| **1^st^ iteration searches** | | | |
| --- | --- | --- | --- |
| **Medline searches**  **QoL terms**  **October 2009** | | | |
| 1 mental health.ti.  2 mental$ ill$.ti.  3 mental$ disorder$.ti.  4 1 or 3 or 2  5 quality of life.ti.  6 4 and 5 | | 1 mental health.ti.  2 mental$ disorder$.ab,ti.  3 mental$ ill$.ab,ti.  4 1 or 3 or 2  5 exp Mental Disorders/  6 4 or 5  7 quality of life.ab,ti.  8 "Quality of Life"/  9 8 or 7  10 6 and 9  11 qualitative research/  12 qualitative.tw.  13 11 or 12  14 10 and 13 | |
| **Medline searches**  **Concepts of possible relevance to QoL**  **October 2009** | | | |
| 1 coping.ti.  2 control.ti.  3 positive attitude$.ti.  4 confidence.ti.  5 anxiety.ti.  6 self-esteem.ti.  7 self-respect.ti.  8 morale.ti.  9 resilience.ti.  10 humo?r.ti.  11 hope$.ti.  12 stress$.ti.  13 (worry or worries).ti.  14 fear$.ti.  15 self-determin$.ti.  16 or/1-15  17 mental$.ti.  18 16 and 17  19 interview$.mp.  20 experience$.mp.  21 qualitative.tw.  22 qualitative research/  23 or/19-22  24 18 and 23 | 1 energy.ti.  2 tired$.ti.  3 physical health.ti.  4 symptom$.ti.  5 hallucinat$.ti.  6 agitat$.ti.  7 paranoi$.ti.  8 (depression or depressive).ti.  9 disinhibit$.ti.  10 ill health.ti.  11 side effect$.ti.  12 sex drive.ti.  13 weight gain.ti.  14 dry mouth.ti.  15 sleep difficult$.ti.  16 substance misuse.ti.  17 substance abuse.ti.  18 gambl$.ti.  19 self harm$.ti.  20 or/1-19  21 mental$.ti.  22 21 and 20  23 interview$.mp.  24 experience$.mp.  25 qualitative.tw.  26 qualitative research/  27 or/23-26  28 27 and 22 | 1 opportunit$.ti.  2 (employment or employed or unemployed or unemployment).ti.  3 occupation$.ti.  4 social activit$.ti.  5 leisure activit$.ti.  6 structur$.ti.  7 routine$.ti.  8 social network$.ti.  9 satisfact$ service$.ti.  10 choice$.ti.  11 autonom$.ti.  12 freedom.ti.  13 responsibilit$.ti.  14 season$.ti.  15 individual$ care.ti.  16 education.ti.  17 money.ti.  18 financ$.ti.  19 income.ti.  20 living condition$.ti.  21 neighbour$.ti.  22 plan$ ahead.ti.  23 uncertain$.ti.  24 or/1-23  25 mental$.ti.  26 25 and 24  27 interview$.mp.  28 experience$.mp.  29 qualitative.tw.  30 qualitative research/  31 or/27-30  32 26 and 31 | 1 stigma$.ti.  2 attitude$.ti.  3 useful$.ti.  4 helpful$.ti.  5 role$.ti.  6 isolat$.ti.  7 alienat$.ti.  8 contact$.ti.  9 avoid$.ti.  10 enjoy$.ti.  11 support$.ti.  12 relationship$.ti.  13 belong$.ti.  14 camaraderie.ti.  15 connect$.ti.  16 (companion$ or company).ti.  17 or/1-16  18 mental$.ti.  19 18 and 17  20 interview$.mp.  21 experience$.mp.  22 qualitative.tw.  23 qualitative research/  24 22 or 21 or 23 or 20  25 24 and 19 |
| **2nd iteration searches** | | | |
| **Multiple database searches (ASSIA, CINAHL, PsycINFO, WOS)**  **QoL terms**  **January 2010** | | | |
| **ASSIA** | | | |
| ((TI=(mental health)) or(TI=(mental$ ill$)) or(TI=(mental$ disorder$))) and(TI=(quality of life)) | | (((TI=(mental health)) or(TI=(mental$ ill$) or AB=(mental$ ill$)) or(TI=(mental$ disorder$) or AB=(mental$ disorder$)) or(DE=("psychiatric disorders" or "adjustment disorder" or "affective disorders" or "organic mood syndrome" or "restlessness" or "seasonal affective disorders" or "akathisia" or "alexithymia" or "anxiety disorders" or "acute stress disorder" or "combat disorders" or "generalized anxiety disorders" or "panic disorders" or "nocturnal panic disorder" or "stage fright" or "phobias" or "acrophobia" or "agoraphobia" or "animal phobias" or "anthropophobia" or "claustrophobia" or "dental phobia" or "dysmorphophobia" or "erotophobia" or "school phobia" or "snake phobia" or "social phobia" or "spider phobia" or "weight phobia" or "posttraumatic stress disorder" or "chronic posttraumatic stress disorder" or "combat related posttraumatic stress disorder" or "postabortion syndrome" or "separation anxiety" or "childhood separation anxiety" or "attachment disorders" or "behaviour disorders" or "attention deficit disorder" or "attention deficit hyperactivity disorder" or "compulsive buying" or "compulsive foraging behaviour" or "conduct disorders" or "disruptive behaviour disorders" or "head banging" or "oppositional defiant disorder" or "cenesthopathy" or "character disorders" or "chronic psychiatric disorders" or "communication disorders" or "autism" or "infantile autism" or "selective mutism" or "confusional states" or "conversion disorder" or "delusional disorders" or "capgras syndrome" or "cotard s syndrome" or "fregoli syndrome" or "litigious delusional disorders" or "demonomania" or "cacodemonomania" or "emotional disorders" or "impulse control disorders" or "insanity" or "koro" or "mania" or "hypomania" or "mass psychogenic illness" or "mental illness" or "neuroticism" or "psychoticism" or "movement disorders" or "neuroleptic malignant syndrome" or "neuroses" or "depersonalization disorder" or "dissociative disorders" or "hypochondriasis" or "neurasthenia" or "obsessive compulsive neuroses" or "transference neuroses" or "personality disorders" or "antisocial personality disorder" or "avoidant personality disorders" or "borderline personality disorder" or "dependent personality" or "depressive personality disorders" or "gender identity disorder" or "histrionic personality disorder" or "identity crisis" or "kleptomania" or "multi impulsive personality disorder" or "multiple personality disorder" or "narcissistic personality disorder" or "passive aggressive personality disorder" or "sadistic personality disorder" or "schizotypal personality disorders" or "selfdefeating personality disorder" or "pervasive developmental disorders" or "asperger s syndrome" or "autistic spectrum disorders" or "childhood disintegrative disorder" or "heller s syndrome" or "rett syndrome" or "pica" or "coprophagia" or "psychogenic aspects" or "psychogenic polydipsia" or "psychoses" or "affective psychoses" or "anhedonia" or "bipolar affective disorder" or "cycloid psychosis" or "depression" or "childhood depression" or "death depression" or "delusional depression" or "maternal depression" or "melancholia" or "parental depression" or "paternal depression" or "postnatal depression" or "refractory depression" or "vascular depression" or "paranoid states" or "alcoholic psychoses" or "korsakoff s syndrome" or "mood incongruent psychoses" or "paranoia" or "querulous paranoia" or "shared paranoid disorder" or "folie a deux" or "puerperal psychosis" or "schizophrenia" or "catatonia" or "chronic schizophrenia" or "paranoid schizophrenia" or "paraphrenia" or "schizophreniform disorder" or "unipolar disorders" or "psychotic mood disorders" or "schizoaffective disorder" or "somatoform disorders" or "body dysmorphic disorder" or "briquet s syndrome" or "somatization disorders" or "thought disorder"))) and((TI=(quality of life) or AB=(quality of life)) or(DE="quality of life"))) and((TI=qualitative or AB=qualitative) or(DE="qualitative research") or(DE="qualitative methods") or(DE="qualitative data") or(DE="qualitative analysis")) | |
| **CINAHL** | | | |
| S6 S4 and S5  S5 TI quality of life  S4 S1 or S2 or S3  S3 TI mental* disorder*  S2 TI mental* ill*  S1 TI mental health | | S13 S9 and S12  S12 S10 or S11  S11 (MH "Qualitative Studies+")  S10 TI qualitative or AB qualitative  S9 S5 and S8  S8 S6 or S7  S7 (MH "Quality of Life+")  S6 TI quality of life or AB quality of life  S5 S1 or S2 or S3 or S4  S4 (MH "Mental Disorders+")  S3 TI mental* disorder* or AB mental* disorder*  S2 TI mental* ill* or AB mental* ill*  S1 TI mental health | |
| **PsycINFO** | | | |
| 1 mental health.ti.  2 mental$ ill$.ti.  3 mental$ disorder$.ti.  4 1 or 2 or 3  5 quality of life.ti.  6 4 and 5 | | 1 mental health.ti.  2 mental$ ill$.tw.  3 mental$ disorder$.tw.  4 exp mental disorders/  5 1 or 2 or 3 or 4  6 quality of life.tw.  7 exp "quality of life"/  8 6 or 7  9 5 and 8  10 qualitative.tw.  11 qualitative research/  12 10 or 11  13 9 and 12 | |
| **WOS** | | | |
| # 6 284 #5 AND #4  # 5 36,217 ti="quality of life"  # 4 44,097 #3 OR #2 OR #1  # 3 5,600 ti="mental* disorder*"  # 2 10,629 ti="mental* ill*"  # 1 28,355 ti="mental health" | | # 20 #19 OR #18 Timespan=All Years  # 19 #16 AND #14 Timespan=All Years  # 18 #15 AND #14 Timespan=All Years  # 17 ti=qualitative Timespan=All Years #  16 ts=qualitative Timespan=1900-2000  # 15 ts=qualitative Timespan=2001-2010  # 14 #13 OR #12 Timespan=All Years  # 13 #11 AND #4 Timespan=All Years  # 12 #5 AND #4 Timespan=All Years  # 11 #9 OR #8 OR #7 OR #6 Timespan=All Years  # 10 ti="mental health" Timespan=All Years  # 9 ts="quality of life" Timespan=1900-1970  # 8 ts="quality of life" Timespan=1971-1980  # 7 ts="quality of life" Timespan=1981-1990  # 6 ts="quality of life" Timespan=1991-2000  # 5 ts="quality of life" Timespan=2001-2010  # 4 #3 OR #2 OR #1 Timespan=All Years  # 3 ts="mental* disorder*" Timespan=All Years  # 2 ts="mental* ill*" Timespan=All Years  # 1 Title=(mental health) Timespan=All Years | |
| **Handsearching and citation searching using key references identified as being of potential relevance through experts and through 1^st^ iteration searches.** | | | |
| **3rd iteration searches** | | | |
| **Multiple database searches (ASSIA, CINAHL, Medline, PsycINFO, WOS)**  **Quasi or indirectly related QoL terms**  **April 2010** | | | |
| **ASSIA** | | | |
| (TI=(recovery or (lived experience) or (subjective experience)) or TI=(coping or adaptation or (life functioning)) or TI=((life changes) or (life satisfaction) or wellbeing) or TI=(well being)) and(TI=((mental health) or (mental$ ill$) or (mental$ disorder$))) | | ((TI=(recovery or (lived experience) or (subjective experience)) or TI=(coping or adaptation or (life functioning)) or TI=((life changes) or (life satisfaction) or wellbeing) or TI=((well being) or lifestyle)) or(AB=(recovery or (lived experience) or (subjective experience)) or AB=(coping or adaptation or (life functioning)) or AB=((life changes) or (life satisfaction) or wellbeing) or AB=((well being) or lifestyle)) or((DE="recovery") or(DE="personal experiences") or(DE="experiences") or(DE="life experiences") or(DE="coping") or(DE="coping strategies") or(DE=("adaptation" or "cognitive adaptation")) or(DE="adaptability") or(DE="life changes") or(DE="life changes") or(DE=("wellbeing" or "emotional wellbeing" or "social wellbeing" or "spiritual wellbeing" or "subjective wellbeing")) or(DE=("psychological wellbeing" or "sense of coherence")) or(DE="lifestyle"))) and((TI=(mental health)) or(TI=(mental$ ill$) or AB=(mental$ ill$)) or(TI=(mental$ disorder$) or AB=(mental$ disorder$)) or(DE=("psychiatric disorders" or "adjustment disorder" or "affective disorders" or "organic mood syndrome" or "restlessness" or "seasonal affective disorders" or "akathisia" or "alexithymia" or "anxiety disorders" or "acute stress disorder" or "combat disorders" or "generalized anxiety disorders" or "panic disorders" or "nocturnal panic disorder" or "stage fright" or "phobias" or "acrophobia" or "agoraphobia" or "animal phobias" or "anthropophobia" or "claustrophobia" or "dental phobia" or "dysmorphophobia" or "erotophobia" or "school phobia" or "snake phobia" or "social phobia" or "spider phobia" or "weight phobia" or "posttraumatic stress disorder" or "chronic posttraumatic stress disorder" or "combat related posttraumatic stress disorder" or "postabortion syndrome" or "separation anxiety" or "childhood separation anxiety" or "attachment disorders" or "behaviour disorders" or "attention deficit disorder" or "attention deficit hyperactivity disorder" or "compulsive buying" or "compulsive foraging behaviour" or "conduct disorders" or "disruptive behaviour disorders" or "head banging" or "oppositional defiant disorder" or "cenesthopathy" or "character disorders" or "chronic psychiatric disorders" or "communication disorders" or "autism" or "infantile autism" or "selective mutism" or "confusional states" or "conversion disorder" or "delusional disorders" or "capgras syndrome" or "cotard s syndrome" or "fregoli syndrome" or "litigious delusional disorders" or "demonomania" or "cacodemonomania" or "emotional disorders" or "impulse control disorders" or "insanity" or "koro" or "mania" or "hypomania" or "mass psychogenic illness" or "mental illness" or "neuroticism" or "psychoticism" or "movement disorders" or "neuroleptic malignant syndrome" or "neuroses" or "depersonalization disorder" or "dissociative disorders" or "hypochondriasis" or "neurasthenia" or "obsessive compulsive neuroses" or "transference neuroses" or "personality disorders" or "antisocial personality disorder" or "avoidant personality disorders" or "borderline personality disorder" or "dependent personality" or "depressive personality disorders" or "gender identity disorder" or "histrionic personality disorder" or "identity crisis" or "kleptomania" or "multi impulsive personality disorder" or "multiple personality disorder" or "narcissistic personality disorder" or "passive aggressive personality disorder" or "sadistic personality disorder" or "schizotypal personality disorders" or "selfdefeating personality disorder" or "pervasive developmental disorders" or "asperger s syndrome" or "autistic spectrum disorders" or "childhood disintegrative disorder" or "heller s syndrome" or "rett syndrome" or "pica" or "coprophagia" or "psychogenic aspects" or "psychogenic polydipsia" or "psychoses" or "affective psychoses" or "anhedonia" or "bipolar affective disorder" or "cycloid psychosis" or "depression" or "childhood depression" or "death depression" or "delusional depression" or "maternal depression" or "melancholia" or "parental depression" or "paternal depression" or "postnatal depression" or "refractory depression" or "vascular depression" or "paranoid states" or "alcoholic psychoses" or "korsakoff s syndrome" or "mood incongruent psychoses" or "paranoia" or "querulous paranoia" or "shared paranoid disorder" or "folie a deux" or "puerperal psychosis" or "schizophrenia" or "catatonia" or "chronic schizophrenia" or "paranoid schizophrenia" or "paraphrenia" or "schizophreniform disorder" or "unipolar disorders" or "psychotic mood disorders" or "schizoaffective disorder" or "somatoform disorders" or "body dysmorphic disorder" or "briquet s syndrome" or "somatization disorders" or "thought disorder"))) and((TI=qualitative or AB=qualitative) or(DE="qualitative research") or(DE="qualitative methods") or(DE="qualitative data") or(DE="qualitative analysis")) | |
| **CINAHL** | | | |
| S17   S12 and S16  S16   S13 or S14 or S15  S15   TI mental* disorder*  S14   TI mental* ill*  S13   TI mental health  S12   S1 or S2 or S3 or S4 or S5 or S6 or S7 or S8 or S9 or S10 or S11  S11   TI lifestyle  S10   TI well being  S9   TI wellbeing  S8   TI life satisfaction  S7   TI life change*  S6   TI life functioning  S5   TI adaptation  S4   TI coping  S3   TI subjective experience*  S2   TI lived experience  S1   TI recovery | | S30   S21 and S26 and S29  S29   S27 or S28  S28   (MH "Qualitative Studies+")  S27   TI qualitative or AB qualitative  S26   S22 or S23 or S24 or S25  S25   (MH "Mental Disorders+")  S24   TI mental* disorder* or AB mental* disorder*  S23   TI mental* ill* or AB mental* ill*  S22   TI mental health  S21   S1 or S2 or S3 or S4 or S5 or S6 or S7 or S8 or S9 or S10 or S11 or S12 or S13 or S14 or S15 or S16 or S17 or S18 or S19 or S20  S20   (MH "Life Style Changes")  S19   (MH "Life Style")  S18   (MH "Psychological Well-Being")  S17   (MH "Personal Satisfaction")  S16   (MH "Attitude to Life")  S15   (MH "Life Change Events")  S14   (MH "Adaptation, Psychological")  S13   (MH "Coping")  S12   (MH "Life Experiences")  S11   (MH "Recovery")  S10   TI lifestyle or AB lifestyle  S9   TI wellbeing or AB wellbeing or TI well being or AB well being  S8   TI life satisfaction or AB life satisfaction  S7   TI life change* or AB life change*  S6   TI life functioning or AB life functioning  S5   TI adaptation or AB adaptation  S4   TI coping or AB coping  S3   TI subjective experience* or AB subjective experience*  S2   TI lived experience or AB lived experience  S1   TI recovery or AB recovery | |
| **Medline** | | | |
| 1 recovery.ti.  2 lived experience.ti.  3 subjective experience.ti.  4 coping.ti.  5 adaptation.ti.  6 life functioning.ti.  7 life change$.ti.  8 life satisfaction.ti.  9 well being.ti.  10 wellbeing.ti.  11 1 or 2 or 3 or 4 or 5 or 6 or 7 or 8 or 9 or 10  12 mental health.ti.  13 mental$ ill$.ti.  14 mental$ disorder$.ti.  15 12 or 14 or 13  16 11 and 15 | | 1 recovery.tw.  2 lived experience.tw.  3 subjective experience.tw.  4 coping.tw.  5 adaptation.tw.  6 life functioning.tw.  7 life change$.tw.  8 life satisfaction.tw.  9 well being.tw.  10 wellbeing.tw.  11 Adaptation, Psychological/  12 Life Change Events/  13 personal satisfaction/  14 or/1-13  15 mental$ disorder$.ab,ti.  16 mental$ ill$.ab,ti.  17 exp Mental Disorders/  18 mental health.ti.  19 18 or 16 or 15  20 19 or 17  21 qualitative research/  22 qualitative.tw.  23 21 or 22  24 14 and 20 and 23 | |
| **PsycINFO** | | | |
| 1 recovery.ti.  2 lived experience.ti.  3 subjective experience.ti.  4 coping.ti.  5 adaptation.ti.  6 life functioning.ti.  7 life change$.ti.  8 life satisfaction.ti.  9 well being.ti.  10 wellbeing.ti.  11 1 or 2 or 3 or 4 or 5 or 6 or 7 or 8 or 9 or 10  12 mental health.ti.  13 mental$ ill$.ti.  14 mental$ disorder$.ti.  15 12 or 13 or 14  16 11 and 15 | | 1 recovery.tw.  2 lived experience.tw.  3 subjective experience.tw.  4 coping.tw.  5 adaptation.tw.  6 life functioning.tw.  7 life change$.tw.  8 life satisfaction.tw.  9 well being.tw.  10 wellbeing.tw.  11 "recovery (disorders)"/  12 "experiences (events)"/  13 coping behavior/  14 adjustment/  15 life changes/  16 life satisfaction/  17 well being/  18 or/1-17  19 mental health.ti.  20 mental$ ill$.tw.  21 mental$ disorder$.tw.  22 exp mental disorders/  23 19 or 20 or 21 or 22  24 qualitative.tw.  25 qualitative research/  26 24 or 25  27 18 and 23 and 26 | |
| **WOS** | | | |
| # 25 #24 OR #23 OR #22 OR #21 OR #20 OR #19 OR #18 OR #17 OR #16 OR #15  # 24 #14 AND #10  # 23 #14 AND #9  # 22 #14 AND #8  # 21 #14 AND #7  # 20 #14 AND #6  # 19 #14 AND #5  # 18 #14 AND #4  # 17 #14 AND #3  # 16 #14 AND #2  # 15 #14 AND #1  # 14 #13 OR #12 OR #11  # 13 ti="mental* disorder*"  # 12 ti="mental* ill*"  # 11 ti="mental health"  # 10 ti="well being"  # 9 ti=wellbeing  # 8 ti="life satisfaction"  # 7 ti="life change*"  # 6 ti="life functioning"  # 5 ti=adaptation  # 4 ti=coping  # 3 ti="subjective experience*"  # 2 ti="lived experience"  # 1 ti=recovery | | #17 #16 AND #15 AND #11 Timespan=2009-2010  # 16 ts=qualitative Timespan=2009-2010  # 15 #14 OR #13 OR #12 Timespan=2009-2010  # 14 ts="mental* disorder*" Timespan=2009-2010  # 13 ts="mental* ill*" Timespan=2009-2010  # 12 ti="mental health" Timespan=2009-2010  # 11 #10 OR #9 OR #8 OR #7 OR #6 OR #5 OR #4 OR #3 OR #2 OR #1 Timespan=2009-2010  # 10 ts=wellbeing Timespan=2009-2010  # 9 ts="life satisfaction" Timespan=2009-2010  # 8 ts="life change*" Timespan=2009-2010  # 7 ts="life functioning" Timespan=2009-2010  # 6 ts=adaptation Timespan=2009-2010  # 5 ts=coping Timespan=2009-2010  # 4 ts="subjective experience*" Timespan=2009-2010  # 3 ts="subjective experience" Timespan=2009-2010  # 2 ts="lived experience" Timespan=2009-2010  # 1 ts=recovery Timespan=2009-2010 | |
| **4^th^ iteration searches** | | | |
| **Handsearching and citation searching using key references identified as being of potential relevance through experts and through 3rd iteration searches.** | | | |
